# Indoor Positioning on Disparate Commercial Smartphones Using Wi-Fi Access Points Coverage Area

**DOI:** 10.3390/s19194351

**Published:** 2019-10-08

**Authors:** Imran Ashraf, Soojung Hur, Yongwan Park

**Affiliations:** Department of Information & Communication Engineering, Yeungnam University, Gyeongbuk, Gyeongsan-si 38541, Korea; ashrafimran@live.com (I.A.); sjheo@ynu.ac.kr (S.H.)

**Keywords:** indoor positioning, Wi-Fi fingerprinting, received signal strength, access points coverage area, smartphone heterogeneity

## Abstract

The applications of location-based services require precise location information of a user both indoors and outdoors. Global positioning system’s reduced accuracy for indoor environments necessitated the initiation of Indoor Positioning Systems (IPSs). However, the development of an IPS which can determine the user’s position with heterogeneous smartphones in the same fashion is a challenging problem. The performance of Wi-Fi fingerprinting-based IPSs is degraded by many factors including shadowing, absorption, and interference caused by obstacles, human mobility, and body loss. Moreover, the use of various smartphones and different orientations of the very same smartphone can limit its positioning accuracy as well. As Wi-Fi fingerprinting is based on Received Signal Strength (RSS) vector, it is prone to dynamic intrinsic limitations of radio propagation, including changes over time, and far away locations having similar RSS vector. This article presents a Wi-Fi fingerprinting approach that exploits Wi-Fi Access Points (APs) coverage area and does not utilize the RSS vector. Using the concepts of APs coverage area uniqueness and coverage area overlap, the proposed approach calculates the user’s current position with the help of APs’ intersection area. The experimental results demonstrate that the device dependency can be mitigated by making the fingerprinting database with the proposed approach. The experiments performed at a public place proves that positioning accuracy can also be increased because the proposed approach performs well in dynamic environments with human mobility. The impact of human body loss is studied as well.

## 1. Introduction

The widespread proliferation of smartphones during the last decade commenced and accelerated location-based technologies to uphold Location-Based Services (LBS). LBS are offered for both outdoor and indoor applications, and precise location information serves are the backbone of such services. An accurate outdoor location can be accomplished with a Global Positioning System (GPS), which can provide an accurate position of up to a few meters [1]. Even so, it is unable to compute an accurate indoor position, on account of its frequency limitation to move through walls, roofs, and other solid objects. Even if GPS can localize a user in the indoor especially when the user is close to windows, that location information may not be accurate enough to serve the standards of indoor localization. This is in view of the fact that signals from satellites are attenuated from interfering objects including walls, roofs, and other similar items, and in many scenarios the location error may be larger than the indoor area itself. Such GPS limitations for indoor positioning necessitated the emergence of alternative indoor positioning and localization technologies.

A number of suitable indoor positioning technologies can be hand-picked including Radio Frequency IDentification (RFID) [2], Vision [3], Pedestrian Dead Reckoning (PDR), geomagnetic field-based positioning [4,5,6,7], Bluetooth, and Wi-Fi, which can serve as a prospective alternative to GPS. However, even these chosen technologies are not without merits. Four potential elements of an Indoor Positioning System (IPS) to define its wide applicability are: deployment cost, ease of use, reliability, and accuracy. Although RFID technology is accurate, it operates at a short distance of a few tens of meters. Even if it less expensive, it requires additional hardware in the form of RFID tags and receivers [8]. Vision-based IPSs do not require any infrastructure and are scalable and cheap. However, IPSs require substantial computational resources to carry out the image matching. Although with modern Graphics Processing Units (GPUs) today’s computers possess enough computational power to do the image matching in real-time, the image matching has its inherent limitations, e.g., image quality, varying light conditions, etc., which limits the wide use of vision-based IPS [9]. PDR systems leverage the data from Inertial Measurement Unit (IMU) and track the user’s position by estimating the traveled distance and heading. However, PDR systems cannot work alone and always require a starting position that they use to predict the next relative position of the user. The user’s movement in complex environments and varying orientations of the smartphone make it even harder to estimate the accurate position of the user. Furthermore, the error in position estimation is accumulated over time if it is not corrected periodically [10].

IPSs, which exploit the Earth’s magnetic (geomagnetic) field, have emerged as an important research area during the past few years. We found a large body of work [11,12] that utilizes the Earth’s natural phenomenon of magnetism to locate a user in the indoor. Such systems utilize the magnetic disturbances in the magnetic field caused by synthetic structures, such as the signatures to locate a user. Despite the fact that these systems are pervasive and require no additional cost, this research area is still in its infancy and substantial effort is required to investigate the attitude of such systems on multifarious devices and in complex environments [13,14]. Bluetooth opened new possibilities for indoor positioning recently. The lower energy power consumption and higher throughput could be very fruitful, especially in the Internet of Things (IoT)-based LBS. Bluetooth IPSs, however, follow a discovery phase where a Bluetooth device scans for other available devices by executing its inquiry protocol [15]. The continuous Bluetooth inquiries drain the battery and increase latency in real-time IPS. Some works to reduce the inquiry time have been presented where the delay is reduced to approximately 4 s [16], but it is still time-consuming and bad for battery life. Additionally, the installation of a large number of Bluetooth devices is required, depending upon the desired level of accuracy and indoor environment complexity.

Wi-Fi-based positioning has been investigated for two decades at least and portrays itself as a strong candidate for indoor positioning. Wi-Fi-based indoor positioning technologies are classified as calibration-free and calibration-based techniques. Calibration-free techniques comprise Time of Arrival (TOA), Time Difference of Arrival (TDOA), Angle of Arrival (AOA), and their variant modifications, which are formed on triangulation and require Wi-Fi signal transmission time and angle. The coexistence of Line-of-Sight (LOS), and Non-Line-of-Sight (NLOS) in hybrid indoor environments, however, restricts the positioning accuracy of calibration-free techniques. Additionally, calibration-free techniques require time synchronization of the receiver/sender side, additional hardware to calculate the angle, and are prone to multipath propagation [17]. The use of Wi-Fi Received Signal Strength (RSS) simplifies the aforementioned problems of time synchronization and angle calculation. Furthermore, as Wi-Fi Access Points (APs) have already been deployed in almost every indoor environment, no cost in terms of additional hardware and infrastructure is incurred. These Wi-Fi IPS are calibration-based systems and are called Wi-Fi fingerprinting systems. The fingerprint pioneered by Bahl in [18] is one of the most popular Wi-Fi positioning approaches today. Fingerprinting refers to the association of various locations in an environment to a particular fingerprint which is unique for that location. In Wi-Fi fingerprinting, a vector which contains the Basic Service Set Identifier (BSSID) and RSS values serves as the fingerprint for a location. Calibration-based Wi-Fi fingerprint IPSs comprise of offline (training) and online (positioning) phases. The offline phase involves the collection of fingerprints at designated locations, normalizing RSS values, and storing them into the database. The online phase, on the other hand, is the positioning process where the user-collected fingerprint is matched against the database to find the user’s approximated position. The positioning phase, however, is affected by many factors including the user’s phone orientation, user’s direction, and the presence of people and obstacles in the environment. Moreover, the use of disparate phones causes huge fluctuations in measured RSS even for the same location at the same time. The above-mentioned factors cause variations in RSS values, resulting in elevated positioning errors. This research aims to solve the pointed out limitations by making the following contributions:

- An indoor positioning approach is presented which solves the problem of device dependence. The proposed approach is tested with three different smartphones including Galaxy S8, LG G6, and LG G7.
- The proposed approach is tested in a static as well as dynamic environment with human presence. The impact of human body loss is evaluated at a public place with low, medium, and high human presence.
- The proposed approach works with different phone orientations in a similar fashion. Also, the testing is performed with user data while walking in different directions.

The rest of the paper is structured as follows. Section 2 describes the limitations of Wi-Fi-based positioning systems. Section 3 overviews the research works related to the current study. Section 4 provides the details of the proposed approach. Section 5 is about the experiment setup, whereas the results are discussed in Section 6. The conclusions are given in Section 7.

## 2. Limitations of Wi-Fi Fingerprinting Positioning

Wi-Fi fingerprinting is one of the most widely used techniques for indoor positioning and have extensively been researched for the last few decades. Even so, it has many limitations, which makes it a potential research area today, and with the widespread proliferation of smartphones equipped with the Wi-Fi sensor and the deployment of Wi-Fi APs in every indoor environment, its importance has further been elevated. This section briefly describes the current issues and limitations of Wi-Fi fingerprinting technique.

### 2.1. Similar RSS at Distinct Locations

One of the root cause of large errors in Wi-Fi signal-based localization is the existence of a similar signature at physically well-separated locations. Wi-Fi signals may have the same RSS values for distinct locations due to the intrinsic phenomenon of the radio signal propagation [19], which may lead to higher errors when localizing a user. This limitation is the fundamental problem of Wi-Fi positioning methods. Such large errors can be overcome and high accuracy, i.e., sub-meter accuracy is achievable but it requires the deployment of hundreds of APs, which is infeasible in practical settings [20].

### 2.2. Impact of Device Diversity

**Figure 1:**
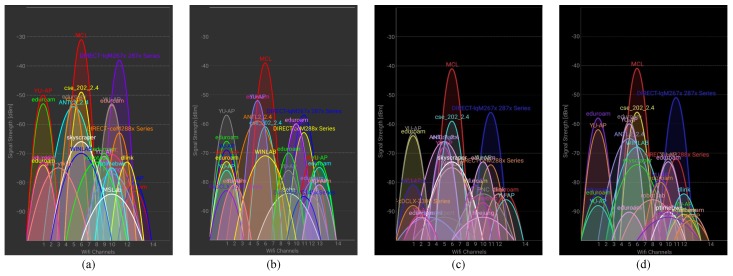
Access Points (APs) and Received Signal Strength (RSS) values using four devices scanned from the same position: (**a**) LG G6, (**b**) LG G7, (**c**) S8 device 1, and (**d**) S8 device 2. Graphs are made using WiFi analyzer [22].

Various Wi-Fi sensors embedded in smartphones are prone to various RSS value while scanned at a given position. Figure 1 shows the graph for scanned RSS values at the same position using Galaxy S8 and LG G6. We can see that the RSS magnitude, even for the same APs at the same point, is different. One noteworthy factor is that the number of detected APs is also different when different user devices are used. Previous research [21], as well as our experiments, revealed that the number of detected APs with different devices is different. Therefore, Figure 1 shows the RSS values of only those APs common for Galaxy S8 and LG G6.

### 2.3. Phone Orientation and User’s Direction

**Figure 2:**
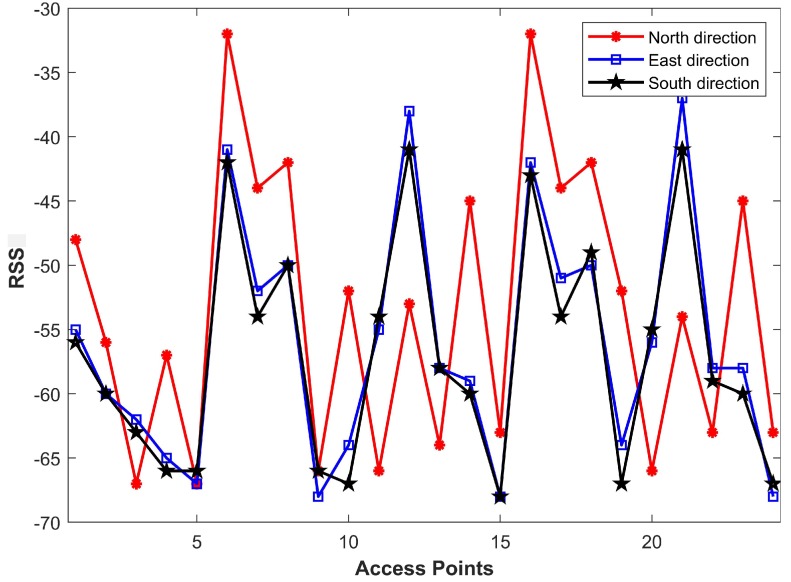
Received signal strength (RSS) values for different Wi-Fi APs in different directions scanned from the same position.

Phone orientation and direction of the user at the time of data collection also affect RSS magnitude. It is observed that RSS may change when RSS is collected while facing different directions. This happens because a direction change affects the LOS of a Wi-Fi AP. Therefore, a LOS AP may become a NLOS AP when we move towards a different direction; for example, from North to South. Figure 2 shows the RSS values collected while facing North, East, and South in the same position with the same smartphone.

**Figure 3:**
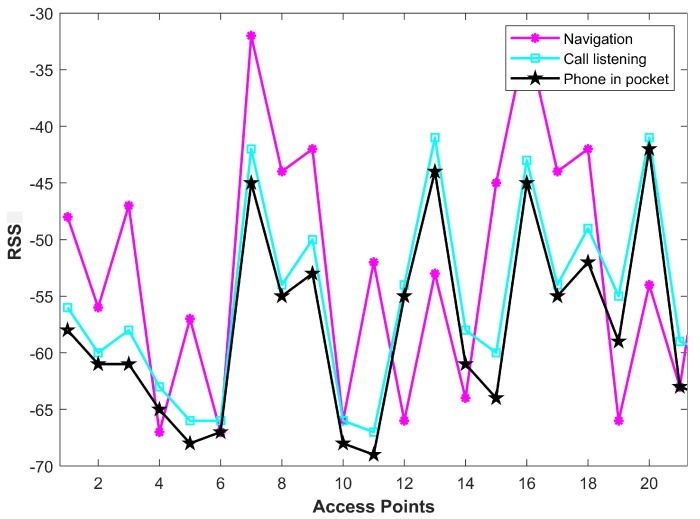
Received signal strength (RSS) values for different Wi-Fi APs with different phone orientations scanned from the same position.

Similarly, when the user changes the orientation of their smartphone, the RSS magnitude scanned from different APs changes as well, even if the direction and the position of the user are not changed. Figure 3 shows the collected RSS graph for users’ different phone orientations including “normal walk”, “call listening”, and “phone in pocket” mode. It shows that the RSS of Wi-Fi APs is vulnerable to change with the change in phone orientation even for the same scanning position. This change in RSS value depends upon user phone holding orientation, and there is no standard way to normalize this value.

### 2.4. Varying Indoor Conditions and RSS

Dynamic indoor environments including the placement of furniture, human mobility, and doors opening and closing cause changes in the collected RSS during the positioning process which increases the localization error [23,24]. Human mobility can cause variations in the observed RSS. As pointed out in [25], the fingerprints collected while the user is not moving to make a less noisy RSS fingerprint. This can potentially improve the positioning accuracy of the system. Besides, real-time positioning involves RSS samples collection while the user is walking, which has a huge impact on positioning accuracy as well.

The human body can influence the propagation of wireless signals as well, which affects the collected RSS [26]. Such RSS variation depends upon the distance between the interfering body and Wi-Fi AP, as well as the count of humans, which interferes with the signals [27]. Additionally, research [28] indicates that the change in temperature of the indoor environment influences the quality of wireless signals, which ultimately causes variation in RSS.

At the same time, Wi-Fi-based localization is highly sensitive to the wall separations and floor plans, so they are vulnerable to random noise, path loss, multipath interference, shadowing, and so on. The authors of [29] point out that the measure signal power from a transmitter contains noise as well. This noise contains several factors including multipath fading, shadowing, antenna pattern, noise, and interference. This noise introduces error in measured RSS and results in erroneous location prediction, because as the RSS variation increases, the predicted positions become less reliable. Similarly, as the shadowing is correlated over a long distance, the location error may rise over to a large area. In the same manner, the authors of [30] suggest that the RSS measurement uncertainty is mainly due to the noise associated with the propagation channel model. Further, it asserts that the uncertainty is dependent on the size of the indoor environment used for positioning. However, in the authors’ opinion, this uncertainty can be reduced with increased number of anchors (APs).

Over and above, Wi-Fi signals are subjected to temporal variations. The authors of [31] indicates that the localization accuracy is higher if the collected test and training data are in close time proximity. However, the localization accuracy falls if the data are collected on a different day [32].

Apart from the above pointed out limitations, RSS-based Wi-Fi systems are vulnerable to environmental dynamics like weather conditions. Such changes can severely downgrade the performance of indoor localization systems, due to the rain caused signal attenuation. The authors of [33] proposed a model to incorporate rain attenuation in the propagation model and enhance the RSS-based distance estimation during rain. Radio propagation parameters and rain rate are estimated offline which can be integrated with real-time RSS to enhance RSS-based distance calculation.

The aforementioned challenges limit the potential and performance of Wi-Fi fingerprinting-based positioning and localization.

## 3. Related Work

Fingerprint-based Wi-Fi positioning technology is undoubtedly one of the most widely used approaches for indoor positioning, due to its cost-effectiveness over other technologies that operate on additionally installed hardware. Pioneered by [18], recent years have seen many improvements to the initial fingerprinting method. However, research is underway to mitigate its various limitations, including the impact of dynamic factors, changes of indoor environment, device dependency, etc. RSS, which is the most popular location fingerprint in fingerprint-based Wi-Fi positioning, is pointed out to vary substantially across different devices even for the same indoor settings. A significant RSS variation is observed due to indoor layout, human mobility, and weather conditions [24]. Two approaches may be applied to reduce the influence of RSS variation on positioning accuracy, whether it is caused by mobility and similar dynamic factors or the inherent limitations of wireless communication. One approach is to enhance and improve the fingerprinting process such that the discrepancy between the offline-collected RSS and user-collected RSS can be minimized. The second approach utilizes other assistive technologies to improve the positioning performance, e.g., the use of pedestrian dead reckoning (PDR), magnetic field positioning, etc.

The authors of [34], for example, investigated the impact of RSS variation on the positioning accuracy and proposed an improved approach to reducing the positioning error. They propose a two-stage positioning algorithm, which works with Wi-Fi fingerprinting. The segment similarity of Wi-Fi APs is used which considers both the RSS value as well as APs. The fingerprint is made of a feature consisting of three segments. Wi-Fi APs are grouped together or split according to the similarity of their RSS value. Experiment output shows promising results. Similarly, the authors of [35] introduced the use of Kullback–Leibler Divergence to calculate the RSS similarity with the Euclidean distance between two location fingerprints to reduce the RSS variance effect of different devices. The results show improved positioning accuracy with various phones.

The authors of [36] investigate the root causes in fingerprint-based positioning responsible for changing the fingerprint of a specific location and propose a method called DorFin. Especially, the RSS inconsistency caused by signal fluctuations and different orientations of commercial smartphones is studied in detail. A discrimination factor is devised that can detect the outlier measurements and reassemble fingerprints to tolerate the transitions. The performance of DorFin is tested in static as well as mobile environments and results are promising. Another noteworthy approach is proposed in [24], which minimizes the impact of various smartphones on positioning accuracy with RSS weights. The weight RSS (WRSS) approach sorts the collected RSS from stronger to weaker and assigns them weights according to their position in the sorted list. Hereby, it stores the fingerprint of a location as a pair where the first element is the RSS value, and the second element is its weight. The WRSS is made in a similar fashion from the user scan and the Euclidean distance is calculated using RSS value and its associated weight when online positioning is performed. The results show that the approach can minimize the impact of different devices on positioning accuracy. The authors of [37] suggest the use of additional parameters for RSS matching to enhance the positioning performance. The features of APs order according to the RSS value, max match count, centroid distance, and entropy error are used.

Despite the fact the improved fingerprinting, as well as positioning algorithm, can substantially lower the Wi-Fi positioning error, researchers tend to work with hybrid approaches to overcome such limitations. For example, the authors of [23] proposed a hybrid approach of radiofrequency and pyroelectric infrared (PIR) sensors. PIR sensors help to identify the zone of the target person and limit the search space. Later a K-nearest neighbor (KNN) approach is applied on Wi-Fi fingerprinting to calculate the exact position of the user.

Similarly, the authors of [38] propose the concept of pairwise signal strength differences (PSSD) to improve Wi-Fi positioning accuracy. The fingerprints are collected as signal strength difference (SSD), rather than RSS fingerprints, to decrease the effect of different devices. Later the topological position of the user is estimated with PDR which helps to further refine the user position.

The process of fingerprinting in Wi-Fi-based IPS is time-consuming and laborious, therefore various research works focus on finding alternatives to traditional surveys. For example, the authors of [39] propose a crowdsensing framework called Bayesian Compressive CrowdSensing (BCCS), which predicts the signal map. The signal map is determined using the correlation between spatial, signal, and temporal dimensions of Wi-Fi APs. The signal map construction is jointly carried out using crowdsensing and Bayesian Compressive Sensing. The proposed framework is able to achieve higher accuracy by 20% to 30% and reduces the data collection cost. BCCS is based on iterative process and accuracy of signal map can be increased with more iterations.

Similarly, the authors of [40] present a fingerprint-based localization system, which can reduce the fingerprinting overhead. The system partitions the area into small regions, where each region is identifiable by the unique AP sequence. After partitioning, each region is assigned a unique AP sequence according to its relative position. Experiment results demonstrate the accuracy of 7.6 m, but sites with thick walls and irregular RSS variation may lead to high error. Similarly, the number of available APs in an area may affect the localization accuracy and lower number of APs lead to worst performance.

Also, Channel State Information (CSI)-based localization systems are investigated to reduce the data collection time and computational resources required for localization. For example, the authors of [41] present a localization system, which is based on Random Forest-based fingerprinting (RFFP), and utilized the CSI information from different Wi-Fi routers. The impact of the various number of features for RF training and testing is also investigated which determines the optimal number of features to achieve higher accuracy. Experiments show that the RFFP system achieves very similar accuracy for LOS and NLOS cases.

The above-mentioned research works are lacking in multiple ways. First, the research works may not have been tested in a dynamic environment, e.g., human mobility and similar other dynamic factors prevalent in real environments. Second, signal depletion over time has not been tested. Third, hybrid systems may require additional infrastructures like the Bluetooth and PIR sensors. Fourth, the fusion of PDR module requires an accurate starting position and longer data from smartphone sensors are needed as well, which increases latency in real-time systems. We aim to build a method that works with Wi-Fi APs alone and can mitigate the impact of device dependency, time-based RSS variation, and dynamic environments.

## 4. Proposed Approach

This section provides the details of the proposed approach, fingerprinting process, and the working principles of the positioning process.

### 4.1. Assumptions for the Proposed Approach

We considered the following assumptions for the proposed approach.

1. The Wi-Fi APs’ position remains the same during the training and testing phases.
2. We assume that the RSS value at a particular location is normally distributed. The same assumption has been made by other works as well [42].
3. To show that the proposed approach is generic by nature and can perform better than other techniques, we have compared its performance with K-nearest neighbor (KNN) found in RADAR [18] and WRSS [24]. The WRSS approach has been chosen because it minimizes device dependency.
4. Another assumption made is that the RSS values of different APs are independent of other APs.

### 4.2. Theoretical Formulations of the Proposed Approach

The proposed approach is founded on two basic principles of Wi-Fi APs, i.e., APs coverage uniqueness and APs coverage overlapping.

#### 4.2.1. APs Coverage Uniqueness

**Figure 4:**
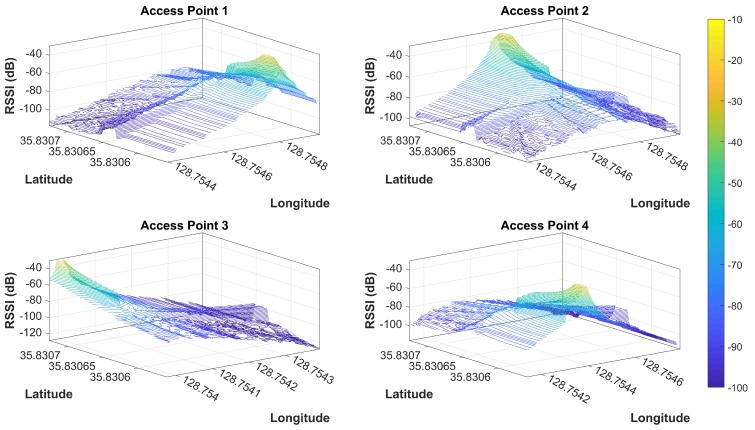
The coverage area of individual APs in the experiment area. The RSS values change due to indoor environment structure.

**Figure 5:**
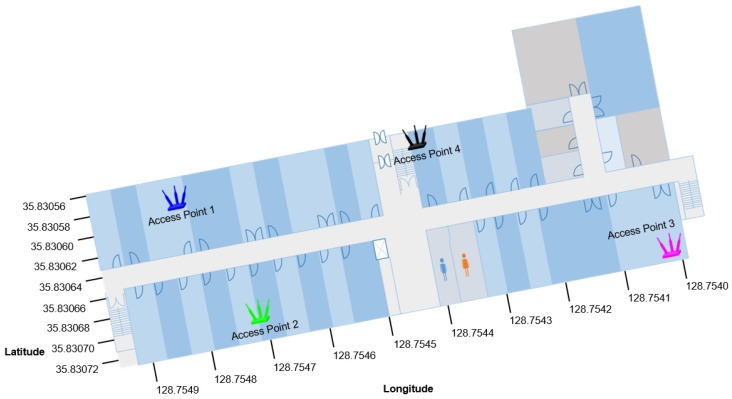
AP locations in the experiment area.

The principle of APs coverage uniqueness implies that every AP has a unique coverage area determined by its fixed location and environment features. The coverage area may be small or large depending upon the AP’s transmission power and other factors, including objects and obstacles present in the environment and other attenuation factors. It also implies that the same AP cannot proved coverage a whole area and that it is not visible/available in a Wi-Fi scan at all locations in a large indoor environment, especially a complex indoor environment. However, it is possible for an AP to be visible at all locations in a small area that does not have walls and similar indoor obstacles. APs coverage uniqueness is denoted by $AP_{ca_{u}}$. Figure 4 shows this concept with the help of plotting a coverage area of four APs located at separate geographic locations on the same floor. The RSS map displayed in Figure 4 is generated with respect to APs locations as shown in Figure 5.

#### 4.2.2. AP Coverage Overlapping

**Figure 6:**
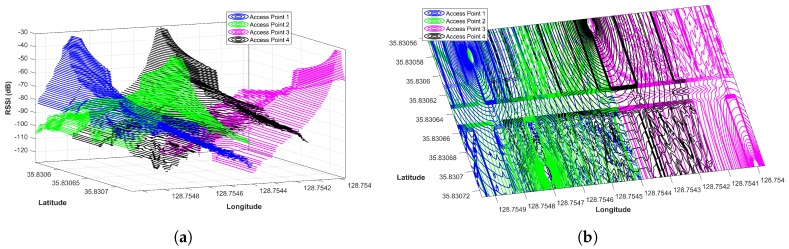
(**a**) AP coverage in the experiment area. (**b**) AP coverage in the experiment area top view.

Contrary to AP coverage uniqueness, AP coverage overlapping implies that in a multiple APs deployed environment, a location may be covered by more than one AP. When we have closely located Wi-Fi APs, an area may be covered by multiple APs at the same time. However, the RSS values of APs may differ for that area, depending on the indoor environment setting. The Wi-Fi AP coverage area that is overlapping is denoted by $AP_{ca_{o}}$ and shown in Figure 6.

**Figure 7:**
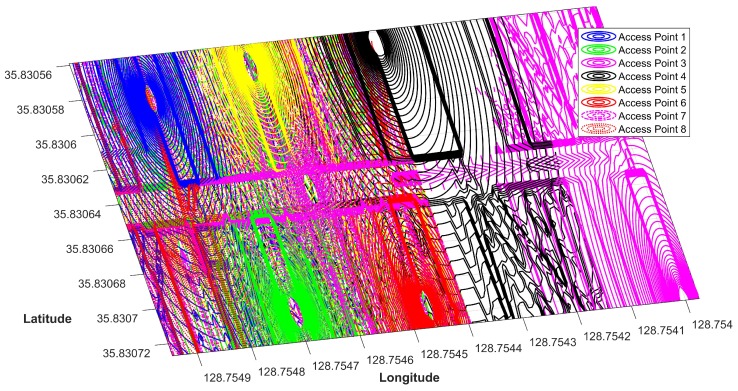
The APs coverage in the experiment area with higher number of APS.

Thus, if the number of APs increases in an area, each point in that area receives signals from a higher number of APs. For example, Figure 7 shows the Wi-Fi coverage from eight APs. Therefore, the intuition is to use the visibility of particular APs at a particular location in the indoor. As when scanned from different devices, the RSS vector may change considerably, in the same way, human mobility and similar other dynamic factors cause substantial fluctuations in RSS values. However, it is observed in our experiments that, in spite of changes in RSS values, the probability of getting similar APs in different device’s scans is high. So, although two devices can get very different RSS values at the same AP, the appearance of the same AP in the scan is highly probable. Unlike traditional fingerprinting methods which utilize RSS vector, our approach is based on the use of appearance of APs at particular locations. We describe the working principles of our approach in the next section.

### 4.3. Fingerprinting Process

The fingerprinting process for our approach is different than that of the traditional approach. The positioning area is divided into a grid, where each grid point is separated by a distance of 1 m. At ground truth points, Wi-Fi scans are made to get the RSS and BSSID of Wi-Fi APs. However, unlike traditional fingerprinting, we do not store the RSS vector. We use the RSS values of APs to filter out weak APs and select good APs only. AP selection is made using the following criteria,

(1) $$\varrho_{i}=\frac{m_{i}}{N}+\frac{\alpha }{ℜ}$$

where $\varrho_{i}$ shows the quality index of $AP_{i}$ and $m_{i}$ is the total number of AP appearances in *N* total number of scans; its maximum can be m=1,2,…,N. We collect five scans at each location to calculate $\varrho_{i}$. The value of *ℜ* in Equation (1) is calculated using

(2) $$ℜ=\frac{1}{m}\sum_{i=1}^{m}RSS_{i}$$

The α in Equation (1) shows the RSS threshold set to calculate $\varrho_{i}$ of a particular AP. Various research works state that −55 dBm to −48 dBm is considered a strong received signal strength indicator (RSSI) average, whereas −79 dBm to −75 dBm is regarded as weak average RSSI [43,44,45,46]. So, considering the values given in the aforementioned research works, we set α as −70 dBm for Equation (1).

The weak APs are discarded with the following standard of ϱ,

(3) $$\left\{\begin{array}{cc} if\;\varrho >=2 & \mathrm{AP}\;\mathrm{selected}\\ otherwise & \mathrm{AP}\;\mathrm{dropped} \end{array}\right.$$

Equation (3) ensures that, to be selected, the AP must have appeared in a higher number of scans, and its RSS should be in the range of strong average RSS.

Our fingerprint database contains only BSSIDs along with the position indices, and no RSS vector is stored. Assume that there are totally *n* number of APs deployed in an indoor environment of a finite space $L=l_{1}\left(x_{1},y_{1}\right),l_{2}\left(x_{2},y_{2}\right),\ldots ,l_{n}\left(x_{n},y_{n}\right)$. The fingerprint vector for location $l_{j}$ can be modeled as $F_{j}=AP_{j1},AP_{j2},\ldots ,AP_{jm}$, where AP refers to the Wi-Fi access point selected using Equation (1) at location $l_{j}$ and *m* shows total number of APs selected through the described criteria at that location.

### 4.4. Positioning Process

The positioning is performed using Algorithm 1. The steps followed in the algorithm are described one by one for clarity:

**Algorithm 1** Algorithm for position estimation**Input:** User scan $W_{S}=\left\{\left(BSSID_{1},RSS_{1}\right),\left(BSSID_{2},RSS_{2}\right),\ldots ,\left(BSSID_{n},RSS_{n}\right)\right\}$**Output:** Position *P*(*longitude*, *latitude*)   *Initialisation*: 1:Sort *S* with respect to *RSS* value. 2:**if***BSSID.RSS* >= *α* **then** 3: add AP to $AP_{S}$ // $AP_{S}$ is selected APs for positioning 4:**end if** 5:**for** each AP *i* in $AP_{S}$ **do** 6: findAllPos($AP_{S_{i}},DB$) 7: **if** DB contains $AP_{S_{i}}$ **then** 8:  add all positions to allAPsPositions 9: **end if**10:**end for**11:Find unique positions and add it to candidatePositions12:**for** all positions in candidatePositions **do**
13: count all occurrences of each candidatePositions in allAPsPositions14:
**end for**
15:Find O=highestOccurenceOfPosition16:Select all positions from candidatePositions whose occurrence is equal to *O*17:Get total number of candidatePositions $P_{c}$18:
**if** $length(P_{c})>7$ **then**
19: calculate spatial proximity $S_{p}$ of each candidate20:
**end if**
21:candidateList = *k* candidates with high $S_{p}$22:Position = GetCentroid(candidateList)23:
**return:** *Position*



The first step in the positioning process is acquiring the user scan, which is collected at an unknown location. The user scan consists of both BSSID and RSS of Wi-Fi APs. The RSS value is used to filter out weak APs. As, during user online positioning, only one scan is collected, Equation (1) cannot be used to calculate ϱ of APs found in the scan. Instead, α is used to remove the weak APs, such that

(4) $$\left\{\begin{array}{cc} if\;RSS_{i}>=\alpha & \mathrm{AP}\;\mathrm{selected}\\ otherwise & \mathrm{AP}\;\mathrm{dropped} \end{array}\right.$$

where $RSS_{i}$ is the signal strength of $AP_{i}$ found in the user scan. The selected APs in this step are denoted by $AP_{S}$ and called selected APs for positioning.

**Figure 8:**
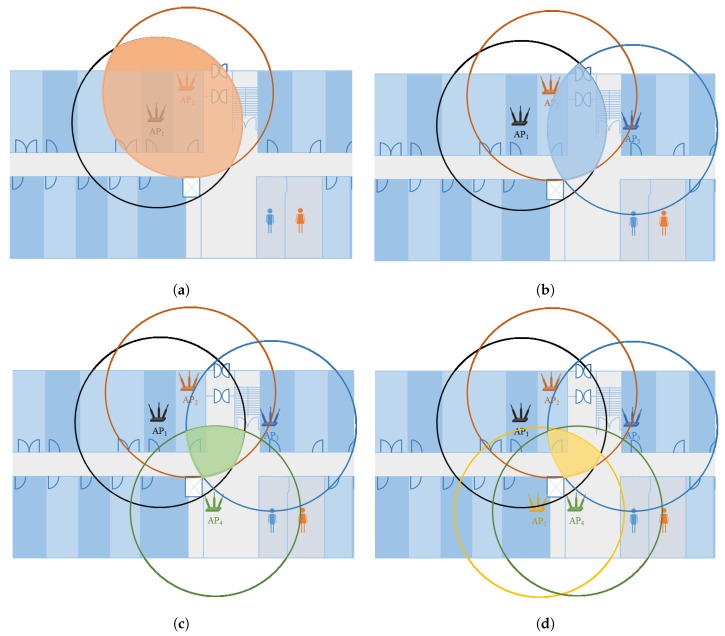
Intersection area based on Wi-Fi APs’ coverage. The coverage area is based on the filtered RSS using α threshold: (**a**) two APs, (**b**) three APs, (**c**) four APs, and (**d**) five APs.

The second step is acquiring the intersected area of APs in $AP_{S}$. As previously described each position in a Wi-Fi deployed area has the coverage from one or more APs. The Wi-Fi coverage area defines the intersection, such that the intersection area decreases as the number of APs increase. This concept is shown in Figure 8. We observe that the intersection is shrinking as the number of APs are increasing in an area. Hypothetically, a higher number of APs coverage in an area will provide a refined position, and vice versa. This is be when the number of APs increases, the intersection area decreases which narrows down the position candidates. However, please note that the given hypothesis does not hold true when we have a very small area of a few meters with a large number of deployed APs.

The position can be calculated on the assumption that

(5) $$\begin{array}{cc} \cap_{a}= & \left\{a:a\in AP_{i}\;\mathrm{and}\;a\in AP_{\left(i+1),(i+2),\ldots ,(i+n\right)}\right\}\\ \; & \;\mathrm{and}\;\cap_{a}\neq \left\{\right\} \end{array}$$

where $\cap_{a}$ represents the intersected area for at least two APs. Equation (5) means that there exists an intersected coverage area where two or more APs signal can be received and their RSS meets the α threshold. If this condition is not met, the user position cannot be established. In this step, we find all positions where APs intersections are found.

During the third step, we need to find the positions where the intersection is high, i.e., the position where a higher number of BSSIDs from $AP_{S}$ and DB are found. However, the practice of selecting a number of positions produces precise results rather than selecting a single position. Keeping in view this approach, we find all positions where APs intersections are same which yields positioning candidates $P_{c}$. One important point to mention is that the number of candidate positions in $P_{c}$ is dynamic and changes with every user request. The candidate positions, $P_{c}$, may be located tightly close or dispersed geographically in some cases. This happens due to human mobility and similar dynamic factors. Dynamic factors lead to the disappearance of a specific AP to positions, which are physically closer and appearance of that AP to a location which is physically well separated. We calculate the spatial proximity of $P_{c}$ candidates to overcome this problem:

(6) $$S_{p}=\frac{1}{min(d_{P_{c}})}$$

where $S_{p}$ is the spatial proximity of a position candidate, and $d_{P_{c}}$ is the distance between a position candidate *i* to all other position candidates. The $d_{P_{c}}$ is calculated using

(7) $$d_{P_{c}}=|P_{c_{i}}-P_{c_{i+1,\ldots ,n-1}}|$$

**Figure 9:**
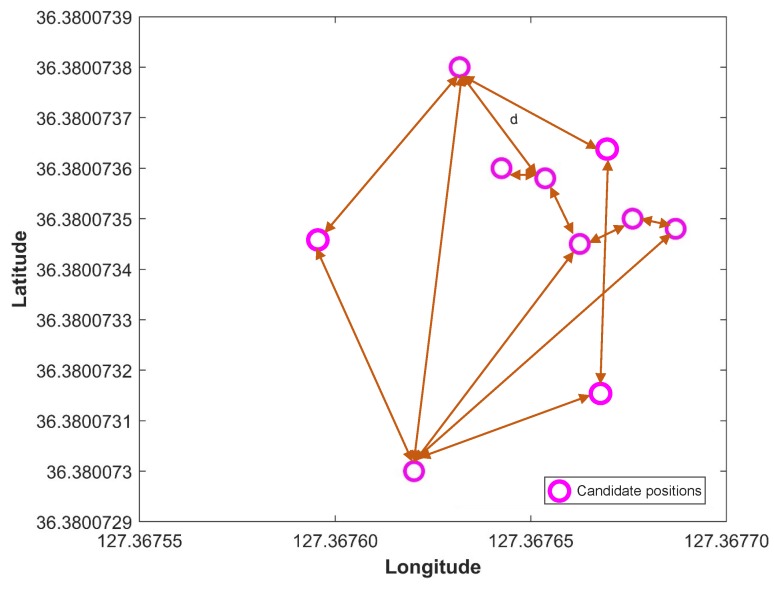
Spatial proximity calculation using the distance between candidate positions.

Figure 9 shows how the spatial proximity is calculated using the position candidates, $P_{c}$. After this step, *k* number of positions with high spatial proximity are selected, where *k* is an empirical value set through the experiments. Our experiments reveal that k=7 proves to be producing higher positioning accuracy.

After calculating the spatial proximity and selected *k* positions from candidate positions, we estimate the user’s current position by calculating the centroid of the selected candidate positions.

## 5. Experiment Setup

**Figure 10:**
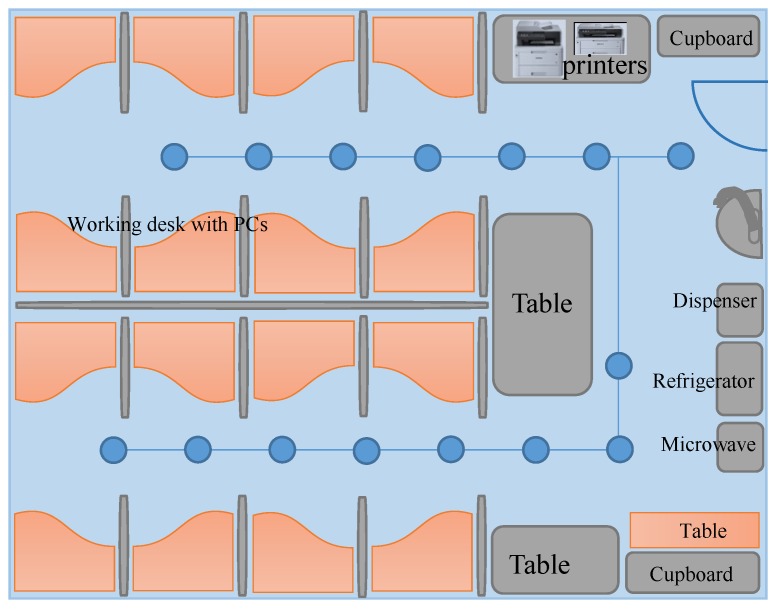
The experiment setup for small room (office environment).

This section describes the experiment set-up and details of the scenarios used in the experiment. The experiment is conducted in Yeungnam University, Korea, using four different buildings with multiple scenarios. Initially, the experiment is performed in a room with an area of 9 × 7 m^2^, as shown in Figure 10.

**Figure 11:**
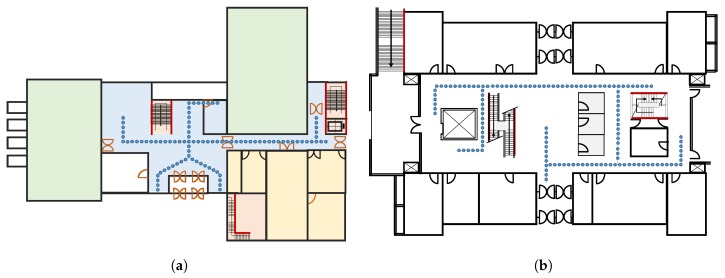
Floor plan of test sites to evaluate the proposed approach. (**a**) CRC building and (**b**) RIC building.

**Figure 12:**
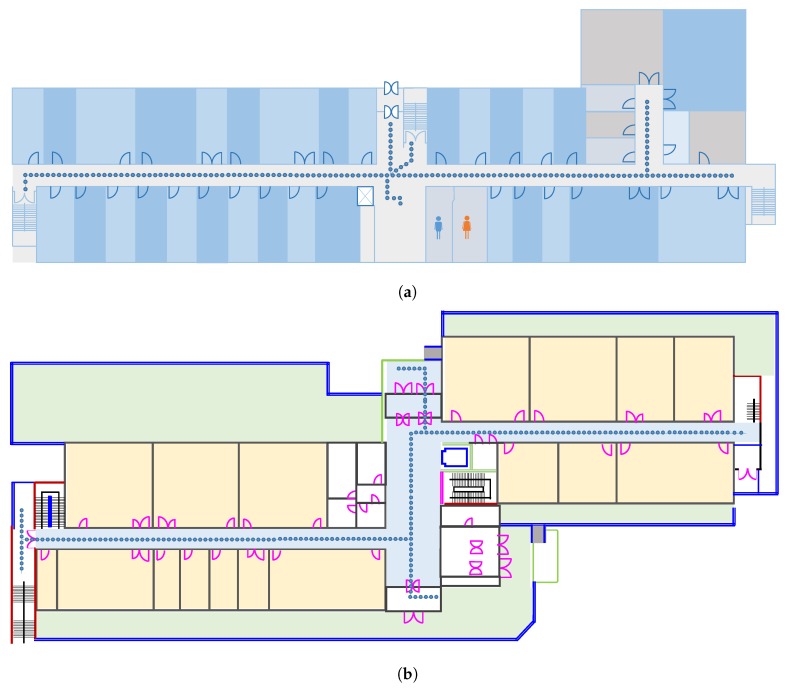
Floor plan of test sites to evaluate the proposed approach. (**a**) IT building and (**b**) TE building.

Figure 11 shows the experiment path followed in the CRC building (Figure 11a) and Regional Innovation Cooperation (RIC) building (Figure 11b), whose dimensions are 70 × 48 m^2^ and 35 × 15 m^2^, respectively. In the same way, Figure 12 shows the testbeds for the Information Technology (IT) building (Figure 12a) and Textile Engineering (TE) building (Figure 12b). The area is 92 × 32 m^2^ and 92 × 42 m^2^ for IT and TE buildings, respectively. Four smartphones are used to conduct the experiments including two Samsung Galaxy S8, an LG G6, and an LG G7 for the evaluation of the proposed approach. One Galaxy S8 is used to survey the environments to prepare the fingerprint database, and the other three phones are used for real-time testing. The fingerprints are collected in a grid, where each point is located at a distance of 1 m. The small circles shown in Figure 11, and Figure 12 represent the points where fingerprints are collected. A total of five Wi-Fi scans at each point are collected to build the fingerprinting database.

## 6. Results and Discussion

This section describes and discusses the results of real-time experiments conducted to evaluate the performance of the proposed approach.

### 6.1. Results for Static Environment

**Figure 13:**
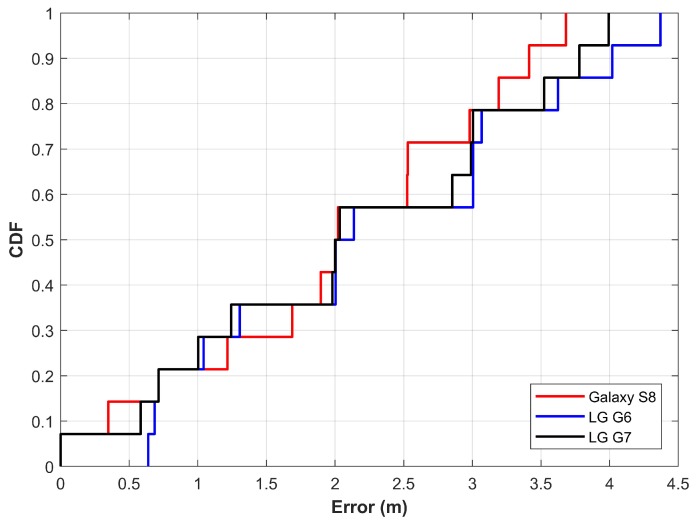
The error graph for room level positioning.

An initial experiment is conducted at a room level to spectacle the efficiency of the proposed approach. The fingerprinting database was built on 22 April 2019, and the experiments are performed during August 2019. Therefore, the time gap between the database formation and the experiment is approximately four months. Figure 13 shows the results of the positioning experiment. The results are quite good considering two important aspects of Wi-Fi positioning.

First, Wi-Fi APs appeared in a smaller area are often same, leading to similar Wi-Fi fingerprints for different positions. Second, the similarity of fingerprint results in the prediction of the same place, even when the user moves to different positions. Both aspects ultimately make it very difficult to predict the user’s position with a lower error. However, the proposed approach performs very well and can achieve an accuracy of 2 m at 50% with heterogeneous devices.

**Figure 14:**
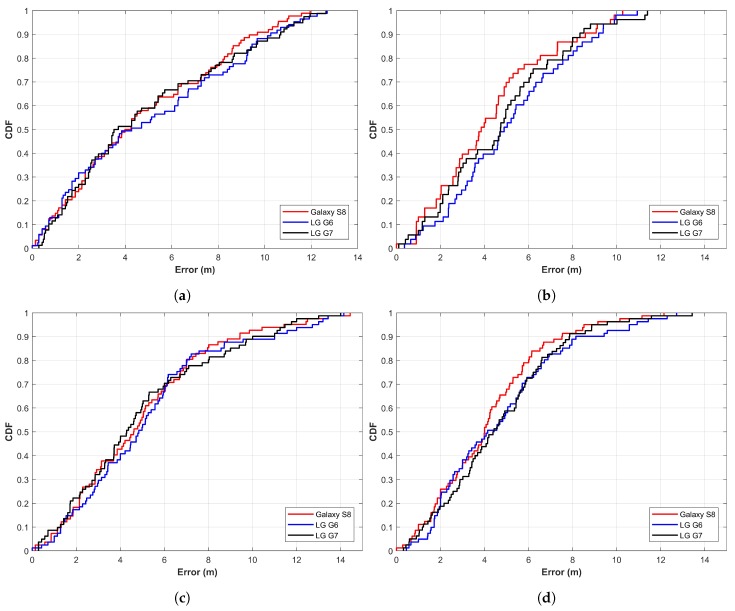
Distance error cumulative distribution function (CDF) for all buildings with all devices using the proposed approach: (**a**) IT building, (**b**) RIC building, (**c**) TE building, and (**d**) CRC building.

Experiments are carried out in larger spaces of four buildings. A different indoor setting as well as different path geometry make the experiment more realistic. Table 1 shows the detailed statistics of the experiment results for each device. Similarly, the cumulative distribution function (CDF) graphs for positioning error are shown in Figure 14.

The experiment results show that the positioning performance of the proposed approach is very similar, even when different models of smartphones are used. Such results are by virtue of the fact that we use the APs coverage area rather than the RSS values. As RSS values can vary significantly from smartphone to smartphone, it may produce very different position prediction. Note that smartphones may show a different list of APs available occasionally, which may lead to different positioning error from different smartphones. However, the experiments prove that the use of APs coverage area feature is more stable than that of APs RSS vector. The distance error graphs shown in Figure 14 prove the same.

**Figure 15:**
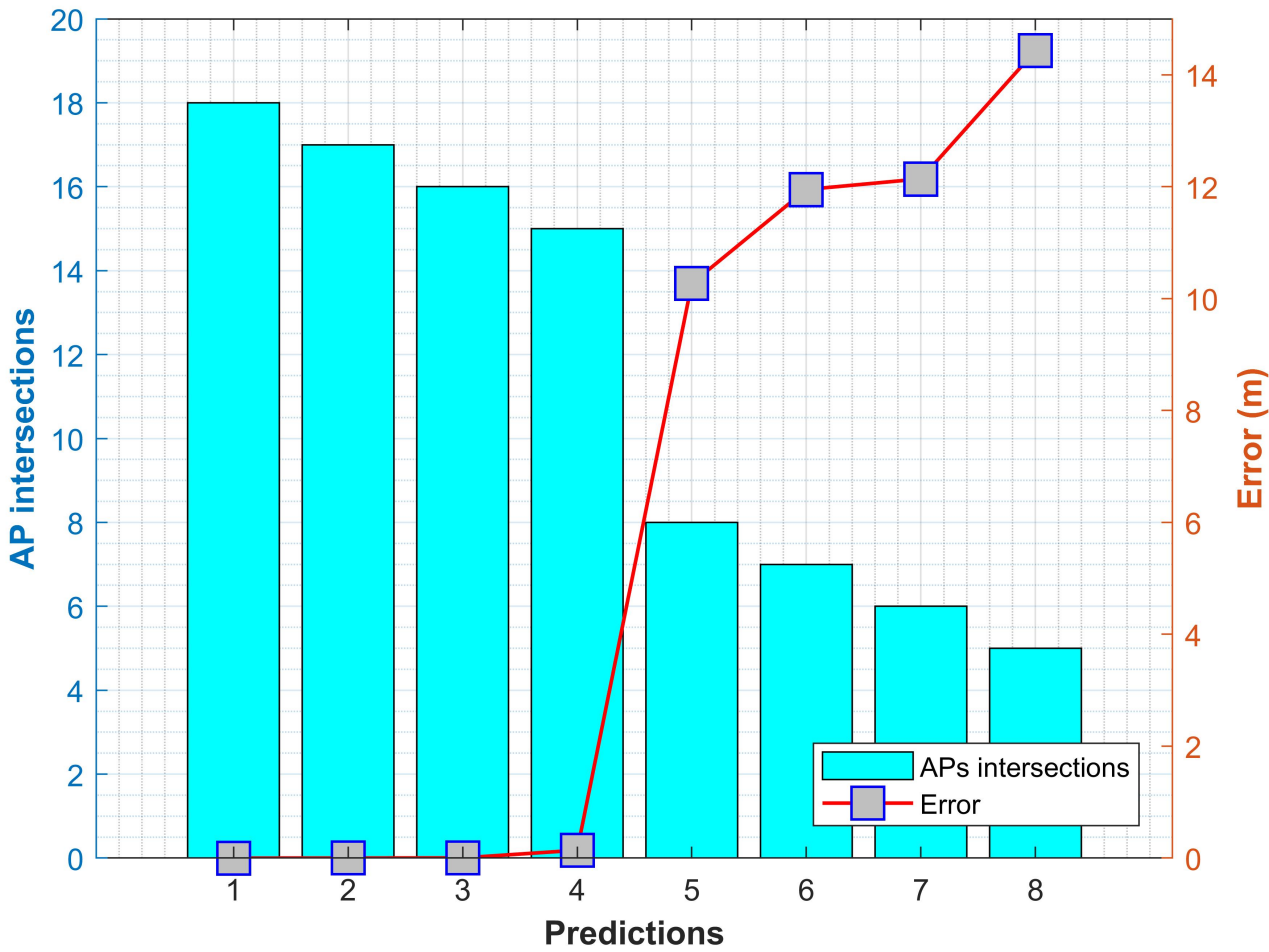
Prediction error against the AP intersection found for the error.

The proposed approach can successfully locate a person within 5 m at 50%, irrespective of the smartphone used for positioning. The mean error and standard deviation prove the effectiveness of the approach by the same token. Our results illustrate that the positioning error for TE and CRC buildings are comparatively higher. Such a difference in error is a consequence of the large and wide space used for positioning. Similar reference fingerprints are observed in these buildings which tend to cause higher errors. An important observation found during the experiments is the impact of the number of visible APs at a particular location on the positioning error. Using fewer visible APs results in higher positioning error and vice versa. IT and RIC buildings have a higher number of APs, and thereby predict more accurate position than those of TE and CRC buildings. As discussed in Section 4.4, using a higher number of APs intersection decreases the position candidates and can predict a more precise position. The experiments prove our hypothesis, as can be seen in Figure 15. The graph shown in Figure 15 is made with the minimum and maximum errors found at each experiment building drawn against the APs intersections for those errors. We can see that a higher number of APs intersections result in minimum errors while the error goes higher when we have a smaller number of intersections. Many research works which use Wi-Fi fingerprint method for indoor positioning narrate that the number of visible/available APs at a particular point has a significant influence on the positioning accuracy. For example, the authors of [47] state that a lower number of APs at a point tends to increase the localization and Root-Mean-Squared Error. Contrary to this, a higher number of APs lead to an accurate position. Likewise, the authors of [40] find that the available APs has substantial impact on the localization accuracy. They further state that the availability of 19 APs ensures an average localization accuracy of 6 m and high. The current study confirms previous findings and Figure 15 shows that in case of 14 or higher number of APs, the average error is below 2 m for the proposed approach. Although the error with high human body loss is higher, the positioning error is still lower than those of other fingerprinting techniques as discussed in the coming section.

### 6.2. Performance Appraisal

**Figure 16:**
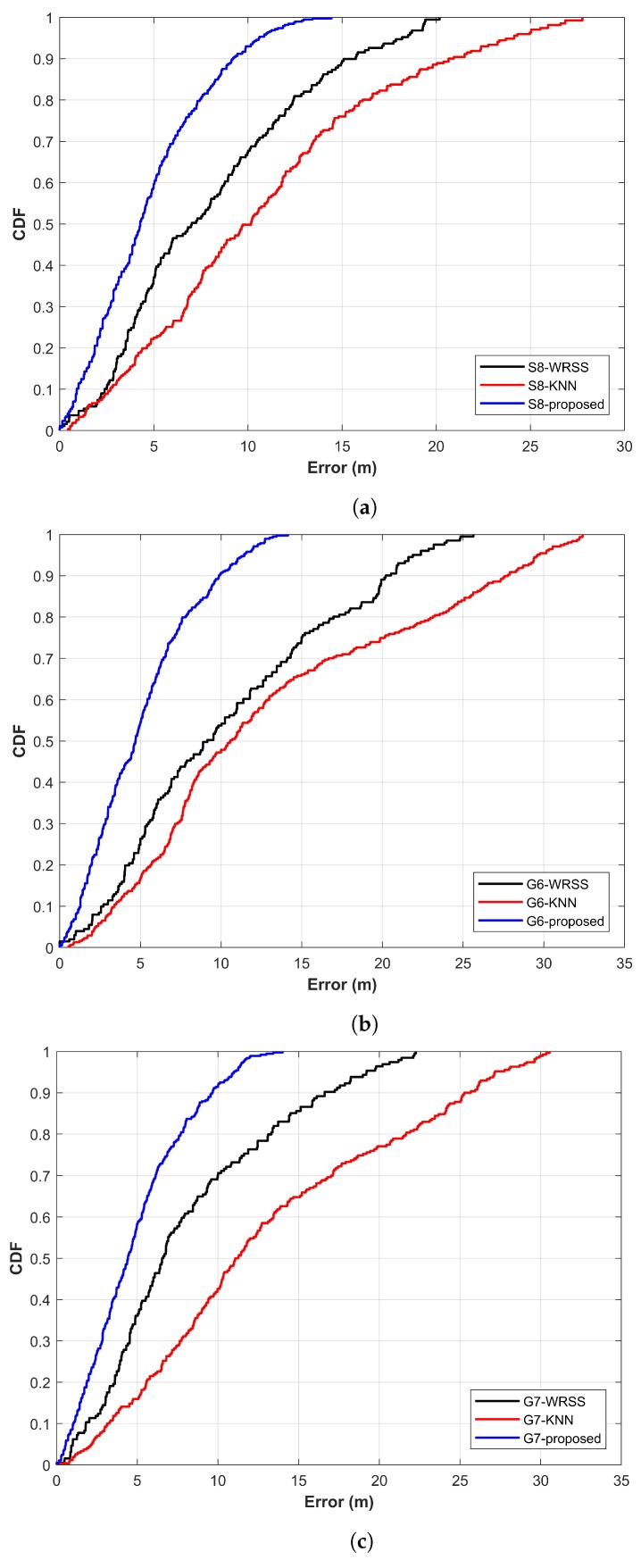
Distance error CDF for all buildings with different techniques using different smartphones. (**a**) S8 smartphone, (**b**) G6 smartphone, and (**c**) G7 smartphone.

The performance appraisal is conducted with two Wi-Fi fingerprinting techniques, including KNN found in RADAR [18] and the WRSS technique [24]. The WRSS technique is proposed to alleviate the impact of device heterogeneity for fingerprint-based positioning and hence we have selected it for performance appraisal. WRSS is implemented for the same environment where the experiments for our proposed approach are carried out. Figure 16 shows the error graphs for KNN, WRSS, and our proposed approach.

The experimental results show that the proposed approach performs better than those of KNN and WRSS for different smartphones. Moreover, the impact of using various smartphones is less conspicuous using the proposed approach. The KNN approach is greatly affected by the use of different models of smartphones. Likewise, WRSS is affected with device heterogeneity and positioning error is different when G6 and G7 smartphones are used, as shown in Figure 16b,c. The positioning errors are higher with KNN and WRSS when the prediction is made using LG G6 smartphone. The error statistics are shown in Table 2.

### 6.3. Results in a Dynamic Environment with Human Presence

The proposed approach is evaluated in a highly dynamic environment at a public place. The Starfield COnvention and EXhibition (COEX) Mall is a famous center situated in Seoul, which contains big halls to organize conferences and exhibitions. The experiments are performed during the Smart Geo Expo 2019 held from 7 to 9 August 2019, when a huge number of visitors attended the exhibition. The experiments are carried out in the exhibition hall area of dimension 108 × 106 m^2^. Experiments are conducted during three different times of the day: morning, noon, and afternoon. The first experiment is undertaken in the morning time when a smaller number of people are present in the exhibition hall. The noontime is attended by relatively a larger crowd and the afternoon hours witness the largest number of people during exhibition days.

The purpose of the experiment is to evaluate the performance of the proposed method in an environment where a large number of people are present and walking freely. Works in the literature [48] prove that the human body poses an effect on the measured RSS in the indoor environment which has a huge impact on the positioning accuracy. The user’s body influences the RSS distribution significantly. The body orientation can change a LOS scenario to NLOS scenario. The body obstructs the signal leading to RSS variation, which makes large positioning errors [42]. The human body affects the signals in two ways: absorption and shadowing and attenuation. Similarly, the authors of [49] show that the presence of people in an indoor environment increases the localization error. Further, the presence of humans may cause distant locations to exhibit very similar RSS signature which drastically affects the localization accuracy [50].

The experiments are conducted at COEX to analyze the impact of human body loss. For this purpose, the scenarios are defined with reference to the number of people present at COEX. The scenarios are characterized as follows.

- Low body loss: 50 to 200 people are present.
- Medium body loss: 201 to 350 people are present.
- High body loss: More than 350 people are present in the area.

**Figure 17:**
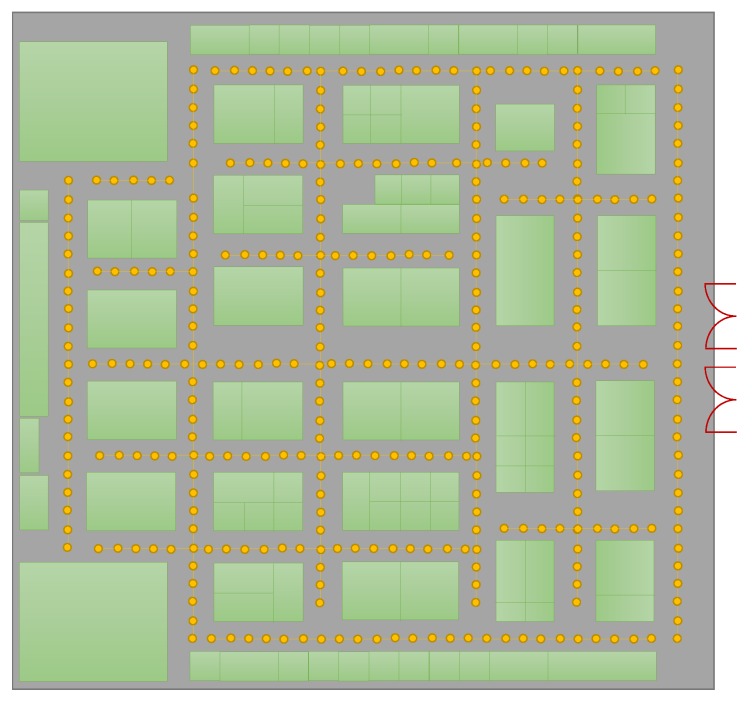
Experiment path used in Starfield COnvention and EXhibition (COEX) center.

The fingerprints are collected with one Galaxy S8 device, and the testing is done with the 2nd S8 device, LG G6, and LG G7. The fingerprints are collected in a grid where each grid point is separated by 3 m distance. Figure 17 shows the paths used for testing. The green squares show the temporary stalls placed from different organizations for the exhibition.

**Figure 18:**
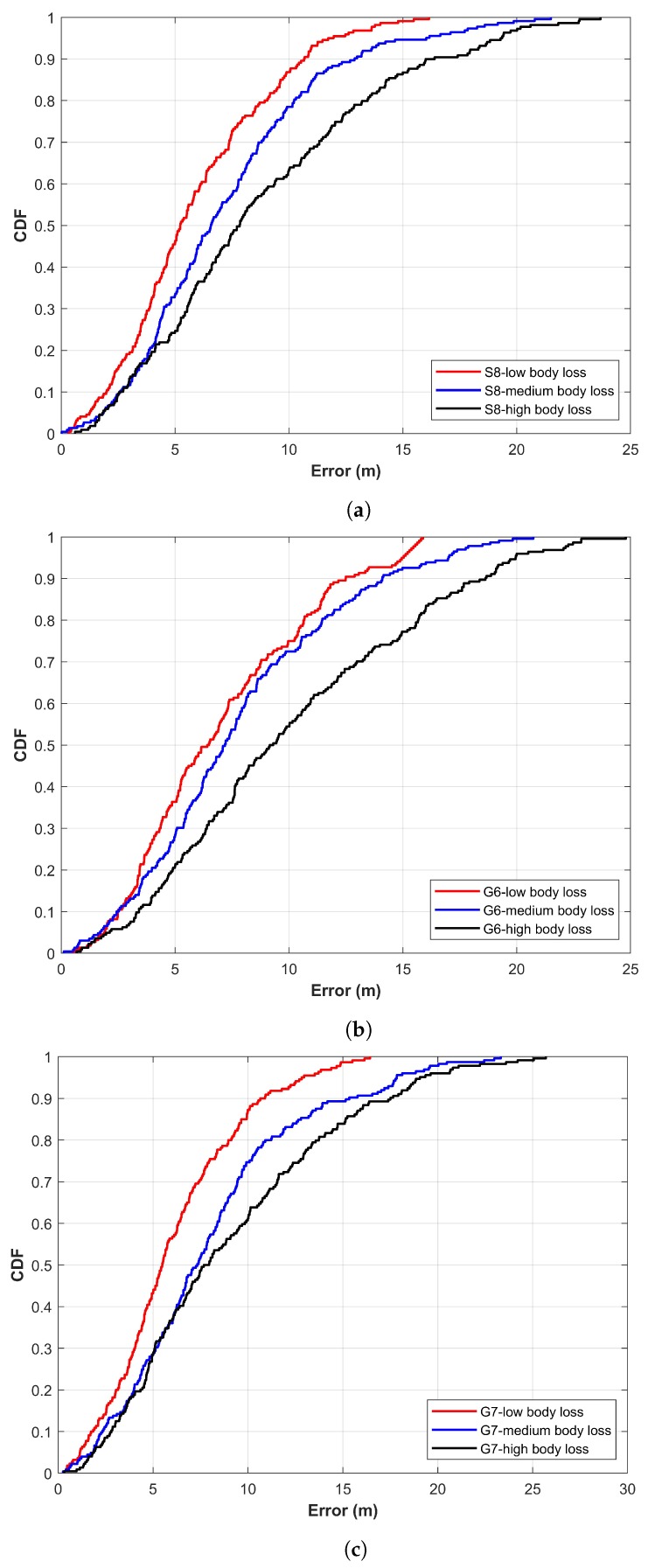
The error CDF for COEX center using different smartphones. (**a**) Galaxy S8 smartphone, (**b**) LG G6 smartphone, and (**c**) LG G7 smartphone.

The positioning results with three different smartphones are shown in Figure 18. Results demonstrate that human presence affects the performance of the proposed approach. The errors for experiment with low human presence are high than those of University buildings experiments. Such errors may pertain to one or many of the following reasons. First, as already explained in the previous section, higher errors are attributed to the large space. The COEX center used for the experiment is larger than University buildings, leading to higher error. Second, many hotspots and portable Wi-Fi APs carried by people walking in and out are found which may not be present during the testing phase. This may lead to a higher error during the user position prediction. Third, COEX center is a high roof building while the University buildings used for the experiment are under 14 feet high. Fourth, even in the presence of low human presence, approximately 100 to 150 people are present, which are moving around in the hall which also degrades the positioning accuracy. Previous research works [50,51,52] show that the human body can substantially change the RSS distribution, which ultimately causes high positioning error. The human body is composed of 70% water which can absorb a part of 2.4 GHz radio signals leading to large signal decay [53]. The RSS loss occurs due to shadowing and attenuation [54]. A standard deviation increase of 2.4 dBm in reported in [42] due to human presence. Regarding the research in localization, [18] reports that the user’s impact on RSS variation is responsible for 67% accuracy degradation.

The positioning accuracy is degraded with medium and high human body loss. The primary reason is the signal absorption, and the secondary reason is multipath interference caused by the human body. Although the proposed approach is not based on the APs’ RSS vector, we use the RSS value to filter out the week APs. Therefore, the human body may lower or variate the RSS value to the set threshold which leads to a lower number of APs intersection and ultimately leads to higher positioning error. Despite this, the mean and maximum errors are not large considering the dimensions of the experiment space and high human presence. The detailed statistics for COEX experiment are shown in Table 3.

### 6.4. Constraints and Limitations of the Proposed Approach

**Figure 19:**
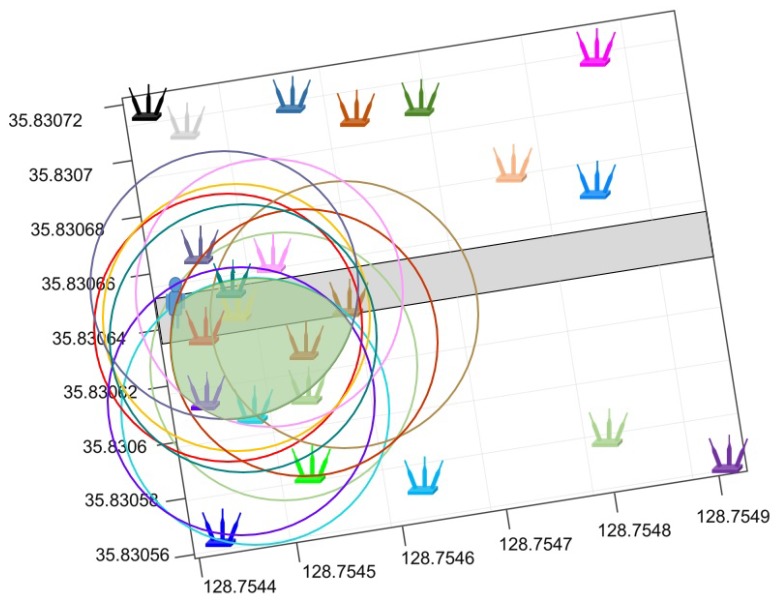
The scenario where APs are geographically close.

**Figure 20:**
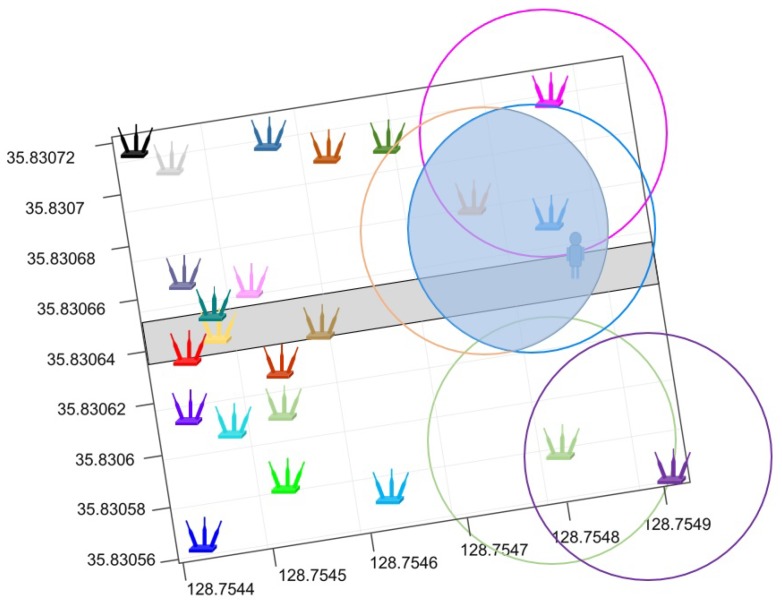
The scenario with geographically scattered APs.

As shown in Figure 15, the number of available APs can dramatically influence the positioning accuracy. The spatial distribution of deployed APs is also taken into account to examine the performance of the proposed method. Two scenarios are of significant importance to this end: large number of APs deployed geographically close to each other and low number of APs distantly scattered. Figure 19 and Figure 20 show the two scenarios, where circles represent the coverage area of a particular AP when its RSS values are higher than −70 dBm.

The experimental results reveal that the positioning error is high when positioning is performed with high and medium human body loss. Such error may be cause by one or more of the following factors. First, the human body causes signal absorption and shadowing which ultimately changes the signal strength. RSS variation, in turn, causes positioning error. Secondly, the proposed approach is based on the APs intersection and overlapping concept, and the hypothesis is that the larger number of available APs at a given point will result in a small intersection area. However, when the APs are geographically closely located, their intersection area may not necessarily be small as shown in Figure 19. Because if the APs are geographically close, their coverage area may be very similar. As a result, the predicted position may have a large error. In the same fashion, if the APs are distantly scattered, there may not be enough intersections to reduce the estimated space which will result in higher positioning error. The authors of [55] point out that a large number of APs should be available to increase the positioning accuracy. This study has the same findings with respect to the impact of APs on the position accuracy.

## 7. Conclusions

This study presents an intuitive approach to alleviating the impact of using heterogeneous devices for Wi-Fi positioning. It improves the accuracy of Wi-Fi fingerprinting without the use of additional hardware. Further, it mitigates the impact of human body loss on positioning accuracy. The proposed approach leverages the use of Wi-Fi APs’ converge area and does not rely on RSS vector, as traditional fingerprinting approaches do. The approach has substantially improved the positioning accuracy of Wi-Fi fingerprinting, especially for public areas which have a high human presence. The real-time experiments reveal that the approach can significantly reduce the device dependence and improve the positioning accuracy across multiple devices. The proposed approach can locate a person within 5 m at 50%, irrelevant of the smartphone used for positioning under static/low mobility conditions. The highest mean errors are 6.45, 7.33 m, and 9.23 m for low, medium, and high human body loss, respectively, with three different smartphones. Future directions include the fusion of the proposed approach with magnetic field data to further refine the accuracy. We believe that using the inertial sensors embedded in the smartphone can considerably increase the tracking and navigation accuracy.

## Figures and Tables

**Table 1:** Positiong error results for all buildings.

Building-Device	Mean Error	Std. Dev	Max. Error	75% Error
IT-S8	4.11	3.35	11.95	7.59
IT-G6	4.27	3.68	12.68	7.69
IT-G7	3.61	3.56	12.63	8.39
RIC-S8	3.85	2.67	10.27	5.54
RIC-G6	4.89	2.71	10.93	7.29
RIC-G7	4.72	2.78	11.40	6.21
TE-S8	4.61	3.20	14.43	6.57
TE-G6	4.84	3.35	14.14	6.72
TE-G7	4.28	3.40	14.00	7.00
CRC-S8	4.00	2.53	12.14	5.69
CRC-G6	4.14	2.91	12.72	6.34
CRC-G7	4.40	2.69	13.43	6.21

**Table 2:** Error statistics using K-nearest neighbor (KNN), weight RSS (WRSS), and the proposed approach.

Building-Technique	Mean Error	Std. Dev	Max. Error	75% Error
S8-WRSS	7.01	5.06	20.17	11.47
S8-KNN	10.16	6.65	27.76	14.59
S8-proposed	4.27	3.10	14.43	6.68
G6-WRSS	9.15	6.61	25.63	14.99
G6-KNN	10.57	8.82	32.42	19.69
G6-proposed	4.79	3.31	14.13	7.21
G7-WRSS	6.57	5.52	22.25	11.62
G7-KNN	11.17	8.04	30.54	18.99
G7-proposed	4.43	3.19	14.01	6.90

**Table 3:** Error statistics for COEX experiment with the proposed approach.

Human Body-Loss	Mean Error	Std. Dev	Max. Error	75% Error
**Galaxy S8**				
Low	5.62	3.59	17.39	7.87
Medium	6.57	4.15	21.50	9.47
High	8.04	5.13	23.68	12.31
**LG G6**				
Low	6.45	3.89	15.88	9.96
Medium	7.19	4.42	20.72	10.57
High	9.23	5.59	24.79	14.55
**LG G7**				
Low	5.90	3.75	17.70	8.50
Medium	7.33	4.83	23.32	10.09
High	7.81	5.51	25.69	12.68
